# Syndecan-1 in the Mouse Parietal Peritoneum Microcirculation in Inflammation

**DOI:** 10.1371/journal.pone.0104537

**Published:** 2014-09-03

**Authors:** Paulina M. Kowalewska, Amanda L. Patrick, Alison E. Fox-Robichaud

**Affiliations:** 1 Medical Sciences Graduate Program, McMaster University, Hamilton, ON, Canada; 2 Thrombosis and Atherosclerosis Research Institute and the Department of Medicine, McMaster University, Hamilton, ON, Canada; Universidade Federal do Rio de Janeiro (UFRJ), Brazil

## Abstract

**Background:**

The heparan sulfate proteoglycan syndecan-1 (CD138) was shown to regulate inflammatory responses by binding chemokines and cytokines and interacting with adhesion molecules, thereby modulating leukocyte trafficking to tissues. The objectives of this study were to examine the expression of syndecan-1 and its role in leukocyte recruitment and chemokine presentation in the microcirculation underlying the parietal peritoneum.

**Methods:**

Wild-type BALB/c and syndecan-1 null mice were stimulated with an intraperitoneal injection of *Staphylococcus aureus* LTA, *Escherichia coli* LPS or TNF*α* and the microcirculation of the parietal peritoneum was examined by intravital microscopy after 4 hours. Fluorescence confocal microscopy was used to examine syndecan-1 expression in the peritoneal microcirculation using fluorescent antibodies. Blocking antibodies to adhesion molecules were used to examine the role of these molecules in leukocyte-endothelial cell interactions in response to LTA. To determine whether syndecan-1 co-localizes with chemokines *in vivo*, fluorescent antibodies to syndecan-1 were co-injected intravenously with anti-MIP-2 (CXCL2), anti-KC (CXCL1) or anti-MCP-1 (CCL2).

**Results and Conclusion:**

Syndecan-1 was localized to the subendothelial region of peritoneal venules and the mesothelial layer. Leukocyte rolling was significantly decreased with LPS treatment while LTA and TNF*α* significantly increased leukocyte adhesion compared with saline control. Leukocyte-endothelial cell interactions were not different in syndecan-1 null mice. Antibody blockade of *β*_2_ integrin (CD18), ICAM-1 (CD54) and VCAM-1 (CD106) did not decrease leukocyte adhesion in response to LTA challenge while blockade of P-selectin (CD62P) abrogated leukocyte rolling. Lastly, MIP-2 expression in the peritoneal venules was not dependent on syndecan-1 *in vivo*. Our data suggest that syndecan-1 is expressed in the parietal peritoneum microvasculature but does not regulate leukocyte recruitment and is not necessary for the presentation of the chemokine MIP-2 in this tissue.

## Introduction

Peritoneal dialysis (PD) is a life-saving replacement therapy for chronic kidney failure. It is estimated that approximately 11% of the worldwide dialysis population of 1.7 million uses PD [1]. In PD, the peritoneal membrane and the underlying microcirculation are used as a dialysis membrane for exchange of solutes and waste products between blood and the dialysis solution. Although PD is an effective renal replacement therapy, technique failure is typically experienced within 6 years of commencement of the therapy [2]. The exposure to dialysis solution drive the deleterious functional alterations of the peritoneal lining and the microcirculation, making it no longer an effective dialysis membrane [3]. Animal studies indicate that the peritoneal catheter may contribute to this pathology as well [4], [5]. In addition, infection and chronic peritonitis exacerbate the peritoneal damage in PD [6]. Thus, the understanding of the molecular mechanisms that drive the histopathology, inflammation and responses to infection of the peritoneal layer is crucial for the development of therapies that can preserve the peritoneum as an effective dialysis membrane.

One of the most common infectious organisms to cause peritonitis in PD is *Staphylococcus aureus* [7]. Interestingly, *S. aureus* was shown to modulate a particular cell surface proteoglycan, syndecan-1 (Sdc1; CD138), to promote its pathogenesis in the corneal tissue [8]. *S. aureus* induced syndecan-1 shedding from the corneal surface and syndecan-1 null mice significantly resisted *S. aureus* corneal infection compared with wild-type animals. Syndecan-1 is a type I transmembrane heparan sulfate proteoglycan composed of a cytoplasmic domain, a transmembrane domain and an extracellular domain containing a proteolytic cleavage site. Syndecan-1 is found on epithelial cell surfaces and is believed to be enmeshed in a structurally complex layer of glycoproteins and proteoglycans known as the endothelial glycocalyx. The major functional domain of syndecan-1 is composed of several heparan sulfate glycosaminoglycan (GAG) chains attached to the distal portion of the extracellular domain of the protein core. The repeating unit of these heparan sulfate chains is a disaccharide of hexuronic acid (either glucuronic or iduronic acid) linked to *N*-acetylglucosamine. Heparan sulfate was demonstrated to bind several different chemokines [9], [10], growth factors [11], coagulation factors [12] and extracellular matrix components [13]. As a result, syndecan-1 plays important roles in many vital processes, including wound repair [14], angiogenesis [15], fibrosis [16], epithelial-mesenchymal transformation [17], [18] and inflammation [10].

Inflammatory reactions are characterized by leukocyte trafficking to and accumulation in inflamed tissues, a process that ensues by 4 hours after the pro-inflammatory stimulus. This involves a multistep process of leukocyte rolling along the endothelial wall, firm adhesion and transendothelial migration [19]. Syndecan-1 was shown to modulate leukocyte recruitment in several different tissues. In the retinal microcirculation, syndecan-1 decreased leukocyte adhesion to the venular wall [20]. In mesenteric venules, absence of syndecan-1 resulted in an increased number of adherent leukocytes and decreased leukocyte rolling [20]. In the lung, syndecan-1 attenuated allergic lung inflammation by suppressing T cell recruitment [21] and generated transepithelial chemokine gradients that mediated neutrophil migration into the alveolar space [10]. In dermal tissue, syndecan-1 attenuated delayed-type hypersensitivity by decreasing leukocyte recruitment [22]. Syndecan-1 was also shown to mediate resolution of neutrophilic inflammation in multiple organs during endotoxemia [23]. These studies suggest that syndecan-1 is a negative regulator of leukocyte adhesion and is involved in generating chemokine gradients during inflammation.

The mechanisms of leukocyte recruitment to the parietal peritoneum, which is perfused by the microcirculation of the musculoperitoneal wall, are not clearly defined. Evisceration studies have shown that the microcirculation of the parietal peritoneum contributes significantly to exchange of solutes in PD [24]–[27]. Therefore, given its significance in PD, the mechanisms of leukocyte recruitment to the parietal peritoneum microcirculation need to be examined. Furthermore, it is not known if syndecan-1 plays a role in peritoneal infection and inflammation. The objectives of this study were to examine the expression of syndecan-1 in the parietal peritoneal lining and microcirculation as well as to determine the role of syndecan-1 in leukocyte recruitment and chemokine presentation in response to *S. aureus* lipoteichoic acid (LTA). To address these objectives, we developed a mouse model of the parietal peritoneum microcirculation using intravital microscopy (IVM). With this *in vivo* technique, we directly visualized syndecan-1 expression and leukocyte-endothelial cell interactions in the parietal peritoneum microcirculation.

## Materials and Methods

### Animals

The animal protocols met the regulations set by the Canadian Council of Animal Care and were approved by the McMaster University Animal Research Ethics Board (Animal Utilization Protocol #11-01-03). Six to eight week old male BALB/c mice were obtained from Taconic (Germantown, NY, USA). The mice were given at least one week to acclimatize. Age-matched syndecan-1 null (*Sdc1* ^-/-^) male mice on a BALB/c background were used in the study. The *Sdc1* ^-/-^ breeders were a kind gift from Dr. Pyong W. Park (Children's Hospital, Boston, MA) and the *Sdc1* ^-/-^ mouse colony was interbred and maintained at McMaster University Central Animal Facility. Under normal laboratory housing conditions, the *Sdc1* ^-/-^ mice are healthy and fertile with normal growth and tissue morphology [28], [29]. The animals were kept in sterilized filter-top cages and housed in a specific pathogen-free facility with a 12-hour light/dark cycle. The mice had free access to water and low-fat rodent chow (Harlan, Teklad, Inc., Madison, WI, USA). The health status of each animal was assessed before experimental procedures.

### Study design

Mice were injected intraperitoneally (IP) with 125* µ*g of LTA from *S. aureus* (Sigma-Aldrich, St. Louis, MO, USA) in 50* µ*L of sterile saline. Lipopolysaccharide (LPS) from *Escherichia coli* serotype 0127: B8 (Sigma-Aldrich, St. Louis, MO, USA) was injected IP at 125* µ*g in 50* µ*L of sterile saline per mouse. Murine recombinant tumor necrosis factor-*α* (TNF*α*) was injected IP at 500 ng in 50* µ*L of sterile phosphate buffered saline per mouse (R&D Systems, Inc., Minneapolis, MN, USA). Control animals were injected IP with 50* µ*L clinical grade saline (0.9% NaCl). All IP injections were done on the right side. Four hours after IP injections of LPS, LTA, TNF*α* or saline, animals were prepared for IVM and the microcirculation underlying the parietal peritoneum was observed.

### Preparation for IVM

The animals were anaesthetized with a subcutaneous injection of a mixture of ketamine (200 mg/kg) and xylazine (10 mg/kg). The subcutaneous route was chosen over IP injection for anaesthetic administration to minimize the disruption of the parietal peritoneum. The fur was clipped over the right ventral neck and the abdomen. The animals were placed on a heat pad and the right internal jugular vein was cannulated with a polyethylene catheter (PE 10, ID 0.28 mm, OD 0.61 mm, Intramedic, Becton, Dickinson and Company, Mississauga, ON, Canada) for maintenance of anaesthesia, administration of fluids or fluorescent antibodies. The skin overlying the abdomen was bluntly dissected away. A midline incision, along the linea alba, was made in the abdominal wall extending inferiorly from the xiphoid process towards the left inguinal region and a flap of musculoperitoneum was created on the left side. Gauze soaked in normal saline was placed over the abdominal contents for constant perfusion of the peritoneum and to keep the abdominal organs intact. The animals were placed in the right lateral position and the flap of peritoneum on the left side of the abdominal wall was laid out on a Plexiglas® microscope stage (Altuglas International, Arkema Inc., Philadelphia, PA, USA). The exposed tissue was immediately covered with plastic wrap (Saran Wrap®; S.C. Johnson and Sons, Inc., Racine, WI, USA) to prevent evaporative loss.

### IVM: fluorescence confocal microscopy

Mice were injected with *S. aureus* LTA (125* µ*g) or saline IP with a dwell time of 4 hours and after preparation for IVM, the left side of the parietal peritoneum was visualized *in vivo*. Fluorescent antibodies were injected via the intravascular cannula and the peritoneal microcirculation was visualized with a spinning disc confocal system (Leica DMI 6000 B; Leica Microsystems, Mannheim, Germany) based on a Yokogawa spinning disc confocal unit, spectral laser merge module LMM5, and Hamamatsu back-thinned EMCCD C9100-12 camera (Hamamatsu, Hamamatsu City, Japan). During the IVM observations, the animals were placed in a chamber (LiveCell2 temperature and CO_2_ environment control system; Quorum Technologies Inc., Guelph, Ontario, Canada) mounted on the confocal microscope and the temperature in the chamber was set to 37°C. The peritoneal microcirculation was observed with a 40× objective lens and images were acquired using the Volocity® 4 acquisition software (Improvision, Waltham, MA, USA), (*n* = 7).

### Reagents for immunofluorescence confocal microscopy

All fluorescent antibodies were injected intravenously (IV) following preparation for IVM 4 hours after IP injection of saline, LTA or TNF*α*. Syndecan-1 was detected with fluorescent monoclonal rat anti-mouse syndecan-1 antibody specific for the extracellular portion of the protein core (40* µ*g, clone 300506; R&D Systems, Inc.). The anti-syndecan-1 antibody was labeled with Alexa Fluor® 488 and Alexa Fluor® 568 Monoclonal Antibody Labeling Kits (Molecular Probes, Burlington, ON, Canada). Alexa Fluor 647-conjugated monoclonal rat IgG1 was used as the isotype control for anti-syndecan-1 (40* µ*g, clone 43414; R&D Systems, Inc.). Platelet endothelial cell adhesion molecule-1 (PECAM-1; CD31) was detected with Alexa Fluor® 647 monoclonal rat anti-mouse CD31 (20* µ*g, clone 390; BioLegend, San Diego, CA, USA).

To examine the expression of adhesion molecules in the parietal peritoneum microcirculation, Alexa Fluor 488-labeled anti-mouse vascular cell adhesion molecule-1 (VCAM-1; CD106) monoclonal rat antibodies (40* µ*g, clone 429 (MVCAM.A); BD Pharminogen, Mississauga, ON, Canada) or anti-mouse intercellular adhesion molecule-1 (ICAM-1; CD54) monoclonal Armenian Hamster antibodies (40* µ*g, clone 3E2; BD Pharminogen) were injected IV 4 hours after LTA challenge. Alexa Fluor 568-labeled rat IgG2a (40* µ*g, clone 54447; R&D Systems, Inc.) was used as the isotype control antibody for anti-VCAM-1 and Alexa Fluor 568-labeled IgG1 (40* µ*g, clone 43414; R&D Systems, Inc.) was used as the isotype control for anti-ICAM-1.

To determine which leukocyte subtypes are recruited in the peritoneal microcirculation 4 hours after saline, LTA or TNF*α* challenge, Alexa Fluor 488-labeled anti-mouse Ly6G/Gr1 monoclonal rat antibodies (40* µ*g, clone RB6-8C5; R&D Systems, Inc.) and Alexa Fluor 568-labeled anti-mouse CX_3_C chemokine receptor-1 (CX_3_CR1) polyclonal goat antibodies (40* µ*g; R&D Systems, Inc.) were co-injected IV.

To examine whether syndecan-1 co-localizes with chemokines *in vivo*, Alexa Fluor 568-labeled anti-syndecan-1 and Alexa Fluor 488-labeled anti-mouse macrophage inflammatory protein-2 (MIP-2; CXCL2) monoclonal rat antibody (40* µ*g, clone 40605; R&D Systems, Inc.), anti-mouse keratinocyte chemoattractant (KC; CXCL1) monoclonal rat antibody (40* µ*g, clone 59526; Creative Diagnostics, Shirley, NY, USA) or anti-mouse monocyte chemoattractant protein-1 (MCP-1; CCL2) monoclonal rat antibody (40* µ*g, clone 123616; R&D Systems, Inc.) were co-injected IV 4 hours after LTA challenge. Alexa Fluor 568-labeled rat IgG2b (40* µ*g, clone 141945; R&D Systems, Inc.) was used as nonspecific isotype control for anti-MIP-2.

To image other members of the syndecan family, Alexa Fluor 488-labeled anti-mouse syndecan-2 monoclonal mouse antibody (40* µ*g, clone F-5; Santa Cruz Biotechnology, Inc., Dallas, TX, USA), anti-mouse syndecan-3 monoclonal rat antibody (40* µ*g, clone 312607; R&D Systems, Inc.) or anti-mouse syndecan-4 monoclonal rat antibody (40* µ*g, clone KY/8.2; BD Pharminogen) were injected IV 4 hours after wild-type mice were injected IP with LTA. Alexa Fluor 568-labeled mouse IgG1 (40* µ*g, clone 11711; R&D Systems, Inc.) or Alexa Fluor 568-labeled rat IgG2a (40* µ*g, clone 54447; R&D Systems, Inc.) were co-injected as isotype control antibodies.

### *Ex vivo* immunofluorescence imaging of the parietal peritoneum

Four hours after LTA treatment, animals (*n* = 3) were anaesthetized and an intrajugular cannula was inserted. Alexa Fluor 488-labeled anti-syndecan-1 (40* µ*g) and Alexa Fluor 647-labeled isotype control antibodies (40* µ*g) were injected IV. Tissue samples of the abdominal wall were collected after 15 min and the animal was euthanized. The tissue samples were embedded in optimal cutting temperature (OCT) compound and snap frozen. The samples were sectioned, counterstained with propidium iodide (PI)-containing mounting medium (Fluoroshield with PI; GeneTex Inc., Irvine, CA, USA) and imaged with a spinning disc confocal system (Leica DMI 6000 B; Leica Microsystems).

### Quantification of fluorescence intensity

The fluorescence intensity of the labeled antibodies was quantified from the captured *in vivo* images of peritoneal venules using ImageJ (NIH, W. Rasband, Bethesda, Maryland, USA). The fluorescence intensity of the Alexa Fluor 488-conjugated anti-syndecan-1 was measured along the length of the basolateral side of the venular endothelium and the value for the corresponding intravascular fluorescence intensity was subtracted. This relative difference in intensity was calculated for 3–4 venules per mouse (*n* = 7) and the values were recorded as the difference in mean fluorescence intensity (gray levels). For each venule, the difference in mean fluorescence intensity between the extravascular and intravascular region was also quantified from the Alexa Fluor 647-labeled isotype control antibodies.

### Syndecan-1 levels measured by enzyme-linked immunosorbent assay (ELISA)

Animals were injected IP with 50* µ*L saline, 125* µ*g *S. aureus* LTA or 500 ng TNF*α*, *n* = 5. Four hours after, the mice were anaesthetized with a subcutaneous injection of a mixture of ketamine and xylazine. Peritoneal lavage was done with 2 mL of clinical grade saline. Blood was collected via cardiac puncture in a heparinized syringe. Peritoneal effluent and blood were centrifuged at 1188×*g*. Samples of the abdominal wall were collected and immediately frozen in liquid nitrogen. The tissues were homogenized in cell lysis buffer (BioVision, Milpitas, CA, USA). Blood plasma and the supernatant from the tissue homogenate were analyzed at a 50-fold dilution. Peritoneal effluent was analyzed at a 10-fold dilution in PBS. Syndecan-1 levels in the tissue homogenate, peritoneal effluent and blood plasma were measured with a commercially available ELISA kit for mouse syndecan-1 (E91966Mu; USCNK Life Sciences Inc., Wuhan, China). To account for differences in tissue sample sizes, a Bradford assay was performed to measure total protein concentration in tissue homogenates and results were normalized to the total protein recovered.

### Transillumination IVM

The microcirculation underlying the left side of parietal peritoneum was observed by an inverted intravital microscope (transillumination technique) (Zeiss Inverted Axiovert 100; Carl Zeiss, Jena, Germany) under a 40× objective lens. Images were captured with an attached camera (Newvicon; DAGE-MTI, Michigan City, IN, USA), projected onto a monitor (Panasonic, CT-2086YD; Panasonic Canada Inc., Mississauga, ON, Canada) and recorded with a DVD recorder (Panasonic, DMR-EH55) for offline analysis. During the intravital observations, the animals were warmed with an infrared heat lamp positioned over the intravital microscope. To minimize the effects of the surgery and exposure of the peritoneal layer, *in vivo* observations were made within 10 min after completion of the surgical preparation for IVM. After completion of *in vivo* imaging, blood was collected into a heparinized syringe via cardiac puncture. Euthanasia was ensured by cervical dislocation.

### Offline analysis

Leukocyte-endothelial cell interactions were quantified in 4–6 venules per mouse (*n* = 4–5) and the number of extravascular leukocytes was determined. Rolling leukocytes were counted per minute and were considered as cells tethering to a venule with torsional motion. Cells that remained stationary for at least 30 seconds were identified as adherent leukocytes. Extravascular leukocytes were counted as perivenular leukocytes on a field of view measuring 180* µ*m ×135* µ*m.

### Leukocyte and differential white blood cell counts

Leukocyte counts were done using a hemocytometer in 0.01* µ*L of blood collected with cardiac puncture immediately following the completion of IVM observations. The blood was mixed with 3% acetic acid and 1% crystal violet in a 5∶44∶1 ratio. Counts were averaged from 6 separate samples. Differential white blood cell counts were performed on 3* µ*L smears of blood fixed in methanol and stained with eosin and thiazine (Harleco Hemocolor stain set; EM Science, Gibbstown, NJ, USA).

### Antibody blockade of adhesion molecules

Wild-type animals were injected IP with anti-mouse *β*_2_ integrin/CD18 monoclonal rat antibody (40* µ*g, M18/2; BioLegend, San Diego, CA, USA), anti-ICAM-1 (40* µ*g, clone 3E2; BD Pharminogen) and/or anti-VCAM-1 (40* µ*g, clone 429; BD Pharminogen). The mice were then stimulated with *S. aureus* LTA (125* µ*g) and after 4 hours, the microcirculation underlying the parietal peritoneum was observed by IVM. For P-selectin (CD62P) studies, animals were prepared for IVM 4 hours after injection of LTA. Baseline recordings were taken and anti-mouse P-selectin monoclonal rat antibodies (20* µ*g, RB40.34; BD Biosciences, Mississauga, ON, Canada) were injected IV through the jugular cannula and the response was recorded after five minutes.

### Statistical Analysis

Data are expressed as mean ± standard error of the mean (SEM). Statistical significance was set at *p*<0.05 and calculated using Student's *t* test or ANOVA with Bonferroni correction for multiple comparisons using the computer software package KaleidaGraph 3.6 (Synergy Software, Reading, PA, USA).

## Results

### Syndecan-1 is expressed in the subendothelial region of post-capillary venules and on the mesothelial layer

**Figure 1:**
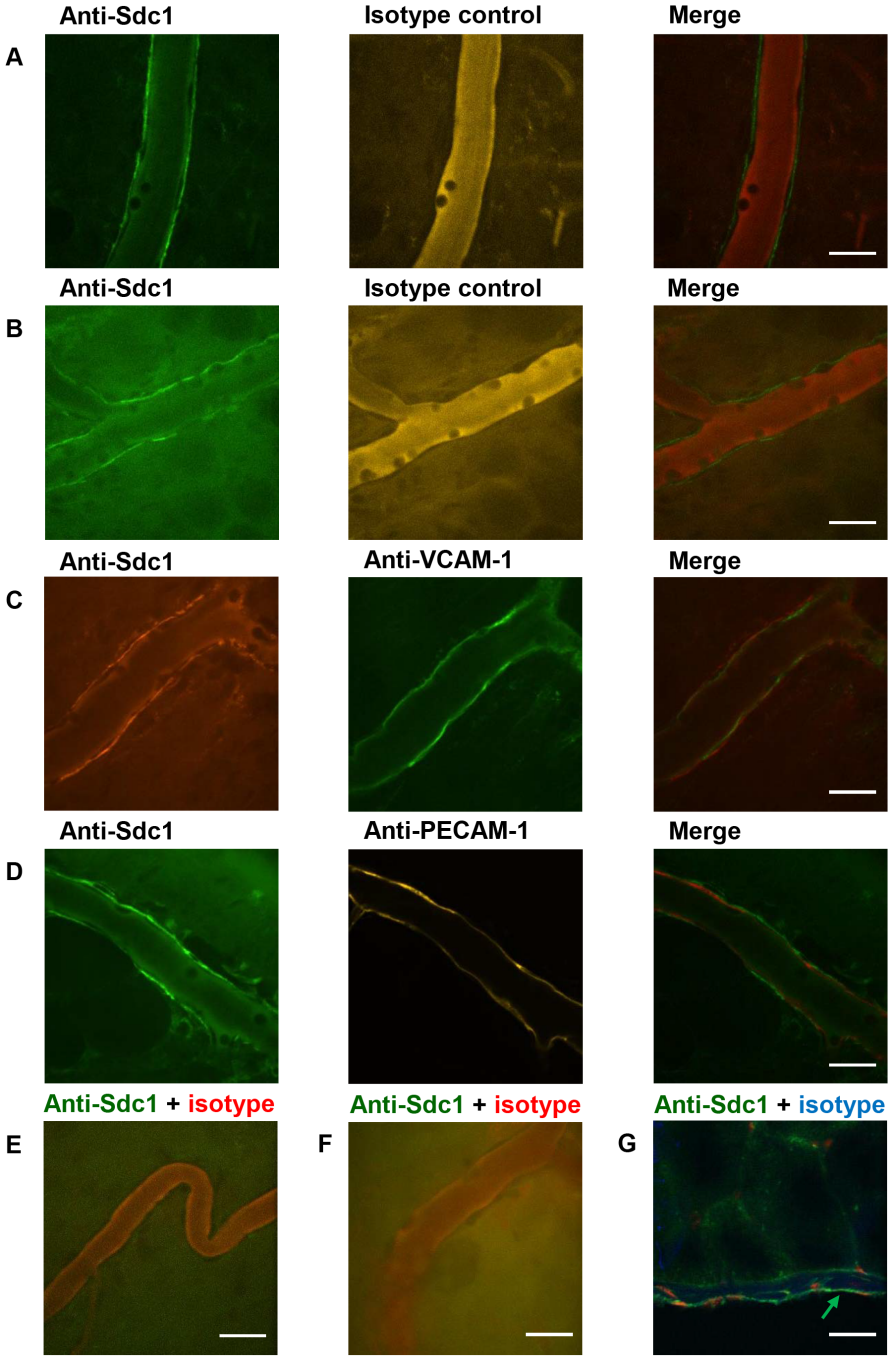
Syndecan-1 is expressed along the basolateral surface of the venular endothelium. *In vivo* fluorescence confocal microscopy images of venules of the parietal peritoneum were acquired and merged after IV injection of Alexa Fluor 488-conjugated anti-syndecan-1 and Alexa Fluor 647-conjugated isotype control antibodies 4 hours after IP injection of (A) sterile saline (50* µ*L) or (B) *S. aureus* LTA (125* µ*g) into wild-type mice, representative images, *n* = 7 mice. Peritoneal venules labeled with (C) Alexa Fluor 568-conjugated anti-syndecan-1 and Alexa Fluor 488-conjugated anti-VCAM-1 or (D) Alexa Fluor 488-conjugated anti-syndecan-1 and Alexa Fluor 647-conjugated anti-PECAM-1, visualized 4 hours after stimulation with LTA, representative images, *n* = 7 mice. Anti-syndecan-1-binding was not detected on (E) arterioles in wild-type animals and (F) venules of *Sdc1* ^-/-^ mice after injection of Alexa Fluor 488-conjugated anti-syndecan-1 and Alexa Fluor 647-conjugated isotype control antibody. (G) *Ex vivo* image of a cross section of the mouse abdominal wall after IV injection of Alexa Fluor 488-labeled anti-syndecan-1 (green) and Alexa Fluor 647-labeled isotype control (blue), counterstained with PI (red), representative image, *n* = 3 mice. Green arrow indicates binding of the fluorescent anti-syndecan-1 to the mesothelium. Scale bar  = 20* µ*m. The *in vivo* and *ex vivo* images showed that syndecan-1 is expressed along the basolateral side of the venular endothelium and is present on the mesothelial lining.

Syndecan-1 expression in the microcirculation that supplies the parietal peritoneum was imaged *in vivo* with fluorescent antibodies after IP injection of saline or *S. aureus* LTA in wild-type mice. The merged fluorescent images showed that while the isotype control antibodies were mostly restricted to the intravascular space, the anti-syndecan-1 antibodies bound to the post-capillary venules away from the lumen in mice injected with saline (**Figure 1A**) and LTA (**Figure 1B**). The small gap between the anti-syndecan-1-labeled surface and the isotype control antibodies in the intravascular space suggests that syndecan-1 expression was localized to the subendothelial region and not the luminal surface. To confirm that syndecan-1 was not expressed on the luminal surface of the venules, wild-type mice were co-injected with fluorescent anti-VCAM-1 and anti-syndecan-1 antibodies. The fluorescence from anti-VCAM-1 appeared on the luminal surface of the endothelium and was mostly distinct from the anti-syndecan-1-bound surface with some small regions overlapping (**Figure 1C**). The venular wall consists of an endothelial layer with a surrounding basement membrane with pericytes. To determine whether syndecan-1 is associated with the endothelial layer, animals were co-injected with anti-syndecan-1 and anti-PECAM-1 antibodies (**Figure 1D**). PECAM-1 is an endothelial cell marker and is concentrated at junctions between cells [30]. Fluorescence from the anti-syndecan-1 antibodies closely followed the anti-PECAM-1-bound layer but only on the basolateral side of the endothelium. Syndecan-1 did not appear to be expressed on the peritoneal arterioles (**Figure 1E**). The specificity of the anti-syndecan-1 antibody was verified in *Sdc1* ^-/-^ mice and no anti-syndecan-1 binding was observed (**Figure 1F**). These findings suggest that syndecan-1 is expressed along the basolateral surface of the venular endothelium.

Since the peritoneal surface could only be viewed in the frontal plane with IVM, it was not possible to determine whether syndecan-1 is expressed on the mesothelial cells that form the parietal peritoneum. Thus, to image the mesothelial layer, transverse sections of the anterior abdominal wall were collected after an IV injection of fluorescent anti-syndecan-1 and isotype control antibodies and imaged with fluorescence confocal microscopy. The *ex vivo* images of the parietal peritoneum revealed that syndecan-1 was expressed on the mesothelial layer (**Figure 1G**).

### Syndecan-1 expression does not change during LTA-induced inflammation

**Figure 2:**
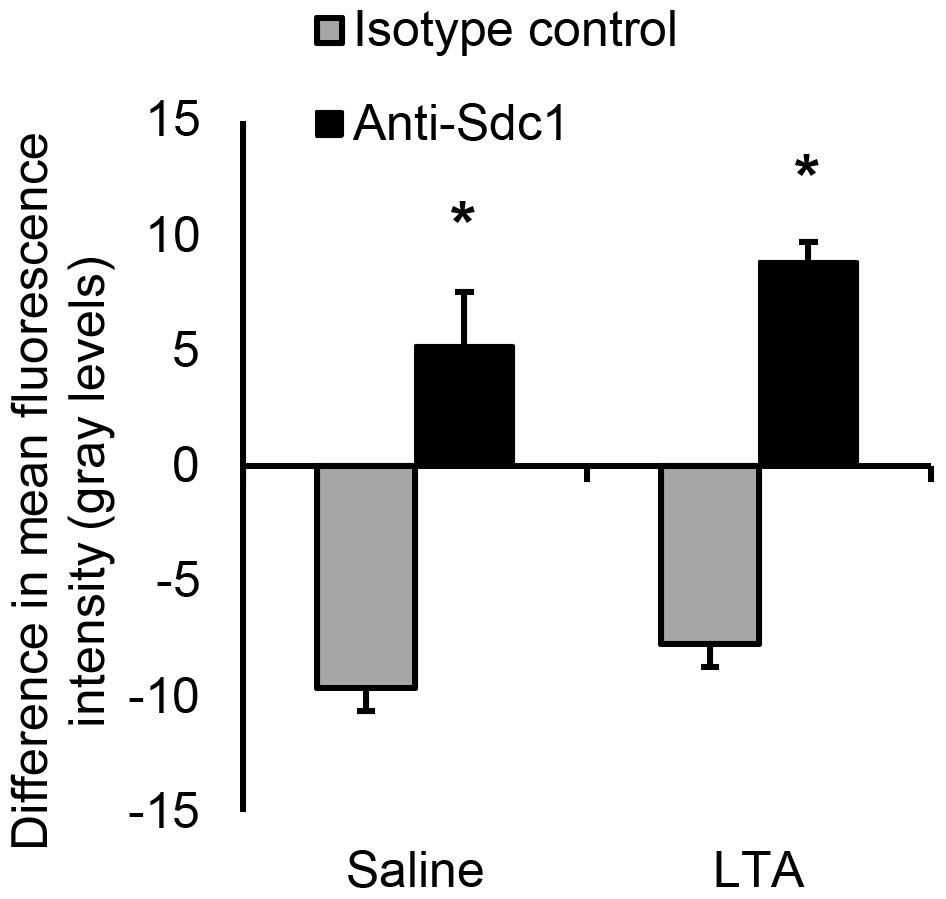
Syndecan-1 expression along the basolateral endothelial surface does not change in inflamed venules. Four hours after stimulation with *S. aureus* LTA (125* µ*g) or 50* µ*L of saline, wild-type mice were prepared for IVM and injected IV with Alexa Fluor^®^ 488-labeled anti-syndecan-1 antibody and Alexa Fluor^®^ 647-labeled isotype control antibody. Images of venules were captured and syndecan-1 expression, reflected as fluorescence intensity of the antibodies that labeled the venules, was measured with image analysis software, ImageJ. The relative values were recorded as the difference in mean fluorescence intensity by subtracting intravascular fluorescence intensity from the fluorescence intensity at the basolateral endothelial surface of a given venule, *p<0.01 compared with isotype control, *n* = 7 mice with 3–4 images analyzed per mouse. Data recorded as mean ± SEM and analyzed with ANOVA with Bonferroni correction. No significant difference in syndecan-1 expression was found between saline and LTA-treated animals.

**Figure 3:**
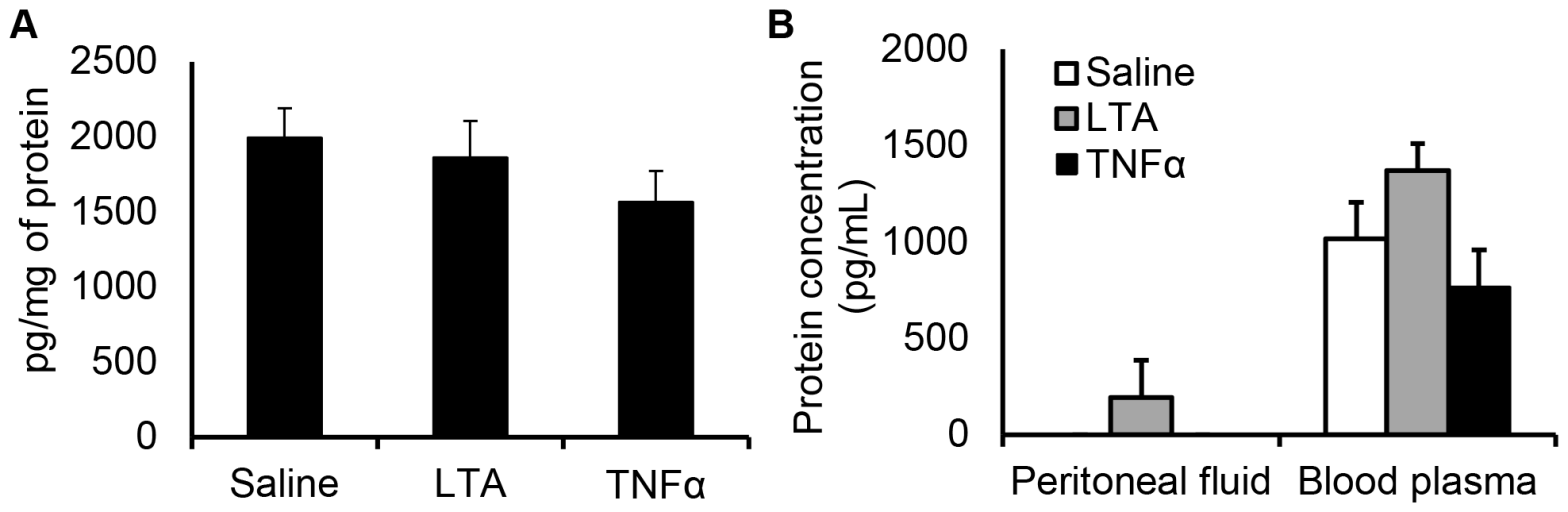
Syndecan-1 expression in the abdominal wall and plasma does not change after LTA injection. ELISA measurements of syndecan-1 levels in (A) mouse abdominal wall samples and (B) peritoneal lavage and blood plasma were taken 4 hours after IP treatment with saline (50* µ*L), LTA (125* µ*g) or TNF*α* (500 ng), *n* = 5 mice. Data recorded as mean ± SEM and analyzed with ANOVA with Bonferroni correction. Syndecan-1 protein levels in the abdominal wall, peritoneal lavage and blood plasma did not significantly differ between saline and LTA or TNF*α*-injected animals.

Because inflammation was shown to alter syndecan-1 levels in tissues [31], the level of syndecan-1 was measured in LTA-induced peritonitis and saline-injected controls. The fluorescence intensity from the syndecan-1 antibodies that bound along the basolateral side of the endothelial layer and the isotype control antibodies was measured using image processing software from the images of venules collected with IVM. The florescence intensity was taken to represent molecular expression on the vascular endothelium. We showed that there is no significant difference in the level of syndecan-1 expression between LTA-treated animals and saline controls (**Figure 2**). This finding was confirmed with an ELISA for syndecan-1. Because TNF*α* was shown to downregulate syndecan-1 expression at the protein and mRNA levels and to promote syndecan-1 shedding *in vitr*o from epithelial cells [31] and also suppressed syndecan-1 expression in endothelial cells [32], syndecan-1 levels were also measured in response to TNF*α* in addition to LTA. The ELISA showed that syndecan-1 levels in the abdominal wall homogenate (**Figure 3A**), peritoneal lavage and plasma (**Figure 3B**) did not significantly differ between mice that were given saline compared to the mice injected with the pro-inflammatory stimuli, LTA and TNF*α*.

### *Sdc1* ^-/-^ mice have normal leukocyte recruitment to the microcirculation underlying the parietal peritoneum

**Figure 4:**
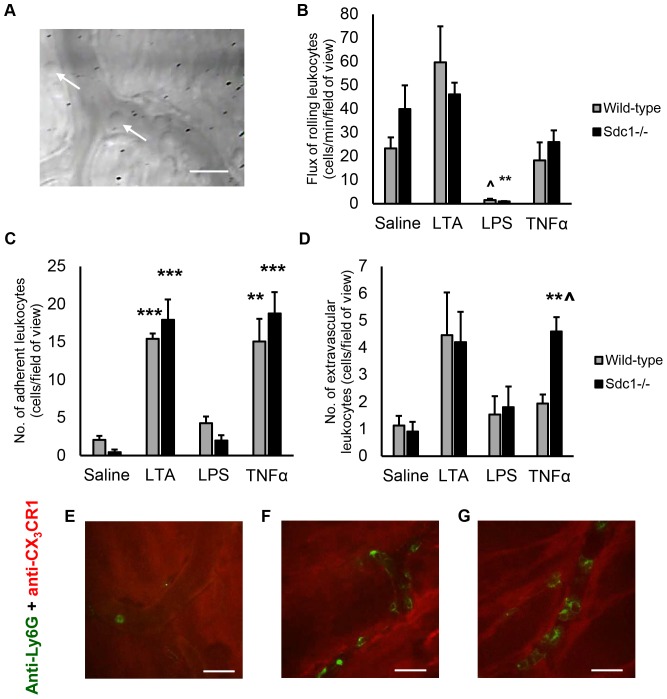
*Sdc1* ^-/-^ mice have normal leukocyte recruitment to the parietal peritoneum microcirculation. Wild-type and *Sdc1* ^-/-^ mice were injected IP with 50* µ*L of saline, *S. aureus* LTA (125* µ*g), *E. coli* LPS (125* µ*g) or TNF*α* (500 ng). Four hours later, the mice were prepared for IVM and (A) the microcirculation of the parietal peritoneum was imaged, arrows indicate leukocytes, scale bar  = 20* µ*m. The number of (B) rolling and (C) adherent leukocytes in the peritoneal venules as well as the number of (D) extravascular leukocytes were quantified. These results show that LPS treatment significantly decreased leukocyte rolling while LTA and TNF*α* treatment significantly increased leukocyte adhesion but the *Sdc1* ^-/-^ mice did not have altered leukocyte recruitment compared to wild-type. Data recorded as mean ± SEM and analyzed with ANOVA with Bonferroni correction, 6 venules averaged per count, *n* = 4 mice, ∧*p*<0.05 compared with wild-type, **p<0.001 compared with saline, ***p<0.0001 compared with saline. *In vivo* fluorescence microscopy images of venules taken 4 hours after administration of (E) saline (50* µ*L), (F) *S. aureus* LTA (125* µ*g) or (G) TNF*α* (500 ng) in mice that were injected IV with Alexa Fluor 488-labeled anti-Ly6G and Alexa Fluor 568-labeled anti-CX_3_CR1, *n* = 3 mice, scale bar  = 20* µ*m. Only Ly6G^+^ cells (neutrophils) were observed in the venules and no intravascular CX_3_CR1^+^ cells (monocytes) were observed at this time.

Leukocyte-endothelial cell interactions were quantified in the peritoneal venules 4 hours after animals were injected IP with *S. aureus* LTA, *E. coli* LPS or TNF*α* using transillumination technique (**Figure 4A**) and the number of rolling, adherent and extravascular leukocytes was compared to saline-injected wild-type controls. IP injection of saline does not alter leukocyte recruitment in the parietal peritoneum microcirculation compared to naïve controls without any injection. Specifically, the numbers of rolling, adherent and extravascular leukocytes in the saline-injected animals were 23.38±4.59 cells/min/field of view, 2.05±0.54 cells/field of view and 1.12±0.36 cells/field of view, respectively, and in the naïve controls, leukocyte rolling, adhesion and extravasation were 27.96±5.52 cells/min/field of view, 1.76±0.40 cells/field of view and 0.84±0.27 cells/field of view, respectively. Leukocyte rolling was significantly decreased with LPS treatment compared with saline controls and did not differ between wild-type and *Sdc1* ^-/-^ mice (**Figure 4B**). The number of adherent leukocytes was significantly increased with LTA and TNF*α* treatment compared with saline controls but did not differ between the wild-type animals and the *Sdc1* ^-/-^ mice (**Figure 4C**). *Sdc1* ^-/-^ mice had a significantly increased number of extravascular leukocytes compared with saline and wild-type controls with TNF*α* treatment (**Figure 4D**). After the completion of IVM observations, cardiac puncture was performed and blood was collected. Total systemic leukocytes were quantified along with differential white blood cell counts and did not show any significant differences between the different treatment groups (**Table 1**). These data suggest that syndecan-1 does not regulate leukocyte recruitment to the parietal peritoneum vasculature and *Sdc1* ^-/-^ mice have normal systemic leukocyte counts.

**Table 1:** Differential leukocyte counts.

Treatment/strain	Total cells ×10^9^/L	Neutrophils	Lymphocytes	Monocytes
**Saline/wild-type**	4.96±1.06	1.12±0.43	3.70±0.81	0.12±0.03
**Saline/** ***Sdc1*** **^-/-^**	6.91±0.89	1.58±0.14	5.25±0.78	0.08±0.01
**LTA/wild-type**	4.10±0.67	2.31±0.31	1.70±0.39	0.09±0.02
**LTA/** ***Sdc1*** **^-/-^**	4.62±0.45	2.86±0.38	1.67±0.34	0.10±0.02
**LPS/wild-type**	1.49±0.25	0.40±0.06	1.03±0.27	0.05±0.02
**LPS/** ***Sdc1*** **^-/-^**	3.29±1.57	0.84±0.29	2.36±1.29	0.08±0.03
**TNF** ***α*** **/wild-type**	3.74±0.59	2.14±0.46	1.55±0.43	0.05±0.03
**TNF** ***α*** **/** ***Sdc1*** **^-/-^**	4.24±0.30	2.31±0.33	1.88±0.06	0.05±0.03

Wild-type and *Sdc1* ^-/-^ mice were injected with saline (50* µ*L), *S. aureus* LTA (125* µ*g), *E. coli* LPS (125* µ*g) or TNF*α* (500 ng). After the completion of IVM observations, blood was collected via cardiac puncture and total systemic leukocyte counts and differential leukocyte counts were performed, *n* = 4 mice. Wild-type and *Sdc1* ^-/-^ mice did not have any significant differences in leukocyte counts in response to the pro-inflammatory agents after IVM.

To determine which leukocyte subtypes are recruited in response to saline, LTA and TNF*α* after 4 hours of exposure, fluorescence IVM of the peritoneal microcirculation was performed after IV injection of Alexa Fluor 488-conjugated anti-Ly6G antibodies to label neutrophils and Alexa Fluor 568-conjugated anti-CX_3_CR1 antibodies to label monocytes. The confocal microscopic images revealed that all of the intravascular cells were Ly6G^+^ while no CX_3_CR1^+^ cells were observed at this time point (**Figure 4E–G**).

### Molecular mechanisms of leukocyte recruitment in the parietal peritoneum microcirculation

**Figure 5:**
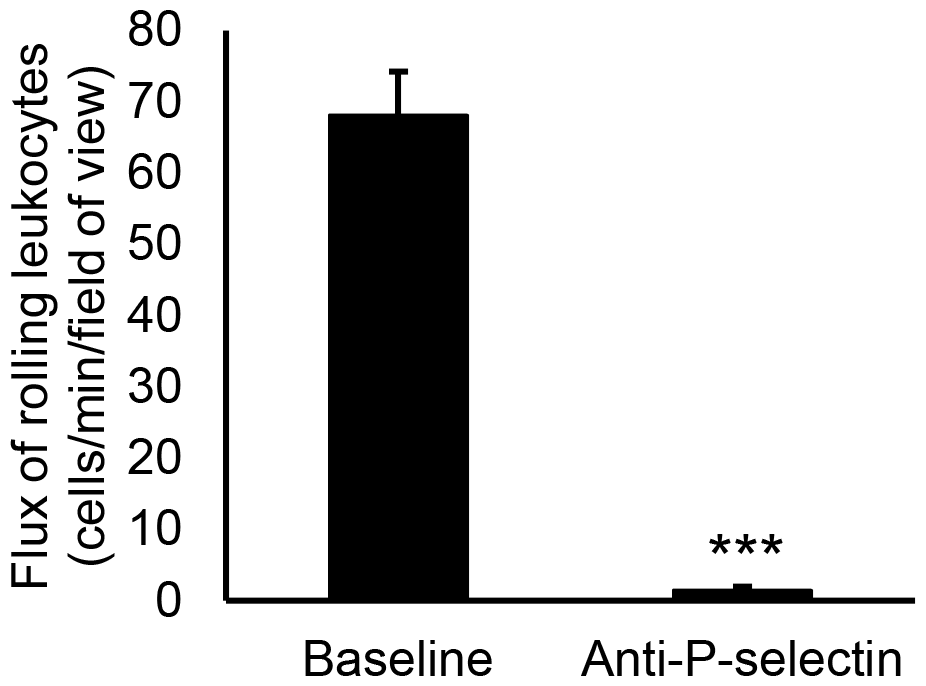
P-selectin mediates leukocyte rolling in the parietal peritoneum microcirculation. Wild-type animals were prepared for IVM 4 hours after injection of *S. aureus* LTA (125* µ*g). Baseline recordings were taken and then anti-P-selectin antibodies were injected IV through the jugular vein cannula and the response was recorded after five minutes. The number of rolling leukocytes in the peritoneal venules was quantified from the recordings. Values for 4 venules were averaged for each mouse with *n* = 4 mice and analyzed with a Student's *t* test, ***p<0.0001. P-selectin blockade inhibited leukocyte rolling in the peritoneal venules in response to LTA.

**Figure 6:**
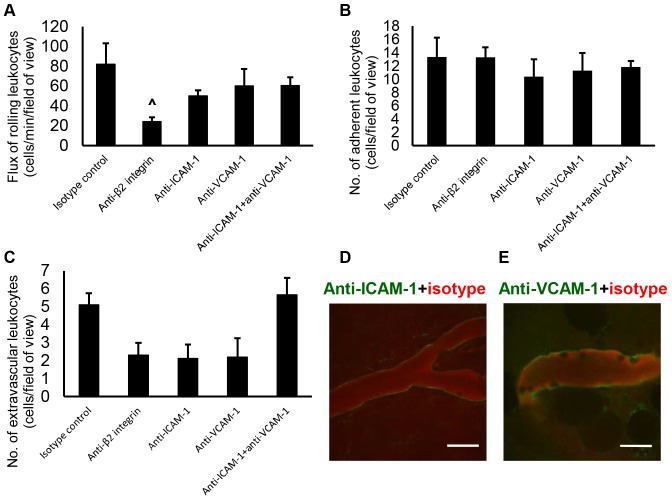
ICAM-1 and VCAM-1 in the parietal peritoneum. Wild-type mice were treated IP with 40* µ*g of anti-*β*_2_ integrin, anti-ICAM-1 and/or anti-VCAM-1 and then challenged with *S. aureus* LTA (125* µ*g). Four hours later, the animals were prepared for IVM (transillumination technique) and the number of (A) rolling and (B) adherent leukocytes in the peritoneal venules as well as the number of (C) extravascular leukocytes were quantified. Data recorded as mean ± SEM and analyzed with ANOVA with Bonferroni correction, 4 venules averaged per mouse, *n* = 4–5 mice, ∧*p*<0.05 compared with isotype control. Expression of ICAM-1 and VCAM-1 in the peritoneal venules was observed with *in vivo* fluorescence confocal microscopy after IV injection of (D) Alexa Fluor 488-labeled anti-ICAM-1 or (E) anti-VCAM-1 with Alexa Fluor 568-labeled isotype control antibodies 4 hours after IP injection of *S. aureus* LTA, *n* = 3 mice, scale bar  = 20* µ*m. Antibody blockade of ICAM-1 and VCAM-1 does not decrease leukocyte adhesion even though both molecules are expressed on the endothelium of the parietal peritoneum microcirculation.

To determine whether P-selectin mediates leukocyte rolling in the parietal peritoneum microcirculation, wild-type animals were prepared for IVM 4 hours after injection of LTA. IVM recordings of venules taken before and after intravenous injection of anti-P-selectin showed that rolling in the peritoneal microcirculation is dependent on P-selectin (**Figure 5**). To identify the molecules that mediate firm adhesion in this tissue, anti-*β*_2_ integrin, anti-ICAM-1 and/or anti-VCAM-1 antibodies were injected IP into wild-type mice immediately before LTA IP injection and IVM was performed after 4 hours. The number of rolling leukocytes was significantly reduced after injection of anti-*β*_2_ integrin antibody due to slower rolling of leukocytes (**Figure 6A**). Treatment with these blocking antibodies did not change the number of adherent leukocytes (**Figure 6B**) or the number of extravascular leukocytes (**Figure 6C**) compared to the isotype control antibody. The finding that blockade of VCAM-1 and ICAM-1 did not decrease the number of adherent cells in the peritoneal microcirculation is surprising as both molecules are expressed on the venular endothelium in this tissue (**Figure 6D–E**). Thus, leukocyte rolling in the parietal peritoneum microcirculation is mediated by P-selectin and anti-*β*_2_ integrin while immunoblockade of ICAM-1 and VCAM-1 does not decrease leukocyte adhesion despite the expression of these molecules on the endothelium of the peritoneal venules.

### MIP-2 expression in the microcirculation of the parietal peritoneum does not depend on syndecan-1

**Figure 7:**
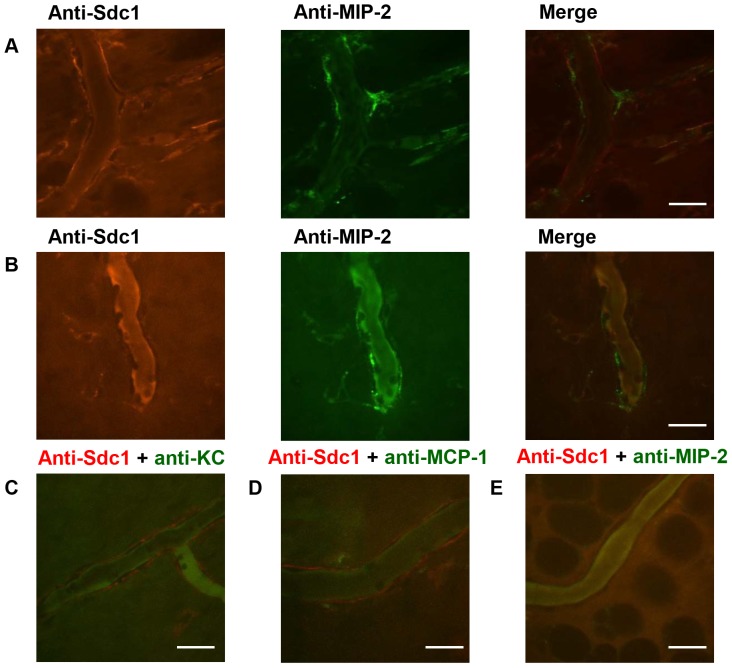
MIP-2 expression in the peritoneal microcirculation does not depend on syndecan-1. Four hours after IP injection of *S. aureus* LTA (125* µ*g), mice were prepared for fluorescence confocal IVM and injected IV with fluorescent antibodies. Microscopic images of MIP-2 expression on the venules of the parietal peritoneum were acquired after IV injection of Alexa Fluor 568-conjugated anti-syndecan-1 along with Alexa Fluor 488-conjugated anti-MIP-2 in (A) wild-type mice and (B) *Sdc1* ^-/-^ mice. No binding of (C) Alexa Fluor 488-conjugated anti-KC nor (D) Alexa Fluor 488-conjugated MCP-1 was detected on the peritoneal venules and (E) Alexa Fluor 488-conjugated MIP-2 did not label arterioles, representative images, *n* = 3 mice, scale bar  = 20* µ*m. MIP-2 is expressed on the peritoneal venules in both, wild-type and *Sdc1* ^-/-^ mice.

To examine whether syndecan-1 co-localizes with chemokines *in vivo*, Alexa Fluor 568-labeled anti-syndecan-1 was co-injected IV with either Alexa Fluor 488-labeled anti-MIP-2, anti-KC or anti-MCP-1 antibodies 4 hours after LTA challenge. Anti-MIP-2 antibody labeled peritoneal venules (**Figure 7A**) in wild-type mice and appeared to partially overlap with the binding pattern of the anti-syndecan-1 antibody. The specificity of the anti-MIP-2 antibody was verified by IV co-injection of Alexa Fluor 488-labeled anti-MIP-2 antibody with Alexa Fluor 568-labeled isotype control, indicating that the binding pattern observed was specific for MIP-2. To determine whether MIP-2 expression on the peritoneal venules is dependent on syndecan-1, *Sdc1* ^-/-^ animals were injected with fluorescent anti-MIP-2 antibodies. IVM imaging revealed that the *Sdc1* ^-/-^ mice have normal expression of this chemokine in the peritoneal venules (**Figure 7B**). Binding of anti-KC (**Figure 7C**) and anti-MCP-1 (**Figure 7D**) was not detected in the peritoneal microcirculation of wild-type animals 4 hours after LTA injection. Anti-MIP-2 and anti-syndecan-1 did not bind to peritoneal arterioles of wild-type animals (**Figure 7E**). These results suggest that at 4 hours after LTA injection, MIP-2 appears in the parietal peritoneum venules but KC and MCP-1 do not. This MIP-2 expression is not dependent on syndecan-1.

### Syndecan-2, but not syndecan-3 nor syndecan-4, is expressed on the venules of the parietal peritoneum

**Figure 8:**
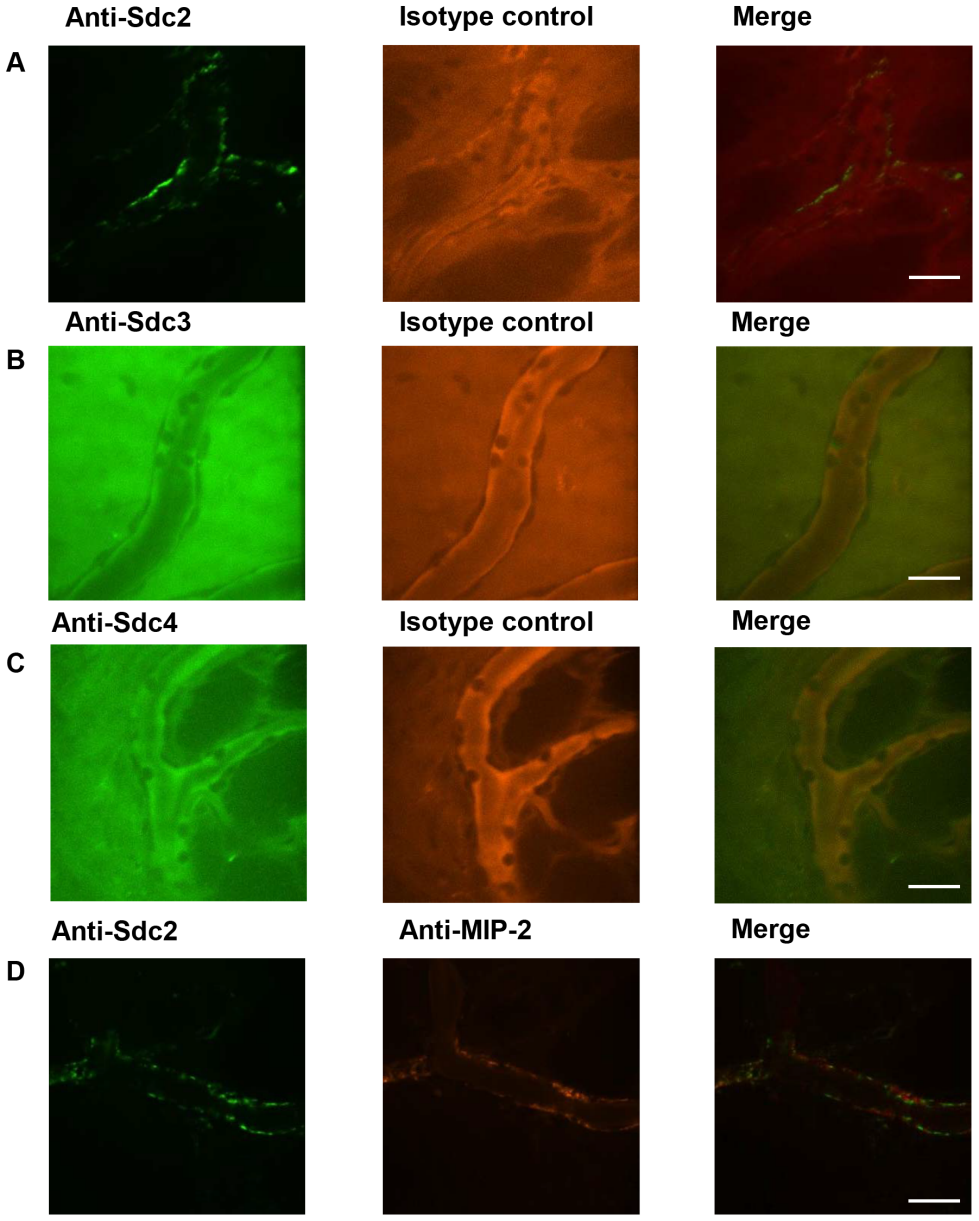
Syndecan-2 but not syndecan-3 nor syndecan-4 is expressed on the peritoneal venules. Wild-type mice were prepared for fluorescence confocal IVM 4 hours after IP injection of *S. aureus* LTA (125* µ*g). (A) Alexa Fluor 488-conjugated anti-syndecan-2 appeared on the venular wall but no binding of (B) Alexa Fluor 488-conjugated anti-syndecan-3 nor (C) Alexa Fluor 488-conjugated anti-syndecan-4 was detected in the peritoneal microcirculation. (D) There was some co-localization between Alexa Fluor 488-conjugated anti-syndecan-2 and Alexa Fluor 568-conjugated anti-MIP-2 in the peritoneal venules. Representative images, *n* = 3 mice, scale bar  = 20* µ*m. Syndecan-2 is expressed on venules and partially co-localizes with MIP-2.

To determine whether there are other heparan sulfate proteoglycans expressed on the peritoneal venules that may bind to chemokines such as MIP-2, wild-type animals were injected IV with Alexa Fluor 488-conjugated anti-syndecan-2, Alexa Fluor 488-conjugated anti-syndecan-3 or Alexa Fluor 488-conjugated anti-syndecan-4 along with Alexa Fluor 568-conjugated isotype control antibodies 4 hours after LTA-stimulation. Syndecan-2 expression was observed on the venules (**Figure 8A**) but no binding of anti-syndecan-3 (**Figure 8B**) nor anti-syndecan-4 antibodies (**Figure 8C**) was detected in the peritoneal microcirculation. There was some co-localization between Alexa Fluor 488-conjugated anti-syndecan-2 and Alexa Fluor 568-conjugated anti-MIP-2 antibodies in the peritoneal venules (**Figure 8D**). These data suggest that syndecan-1 and syndecan-2 may be redundant proteoglycans in chemokine presentation.

## Discussion

Syndecan-1 is believed to be a constituent of the endothelial glycocalyx, a glycoprotein and proteoglycan-rich layer that coats the luminal surface of blood vessels [33]. Since increased plasma levels of syndecan-1 are associated with glycocalyx breakdown, syndecan-1 is used as a marker of glycocalyx degradation [34]. The present study showed that syndecan-1 is expressed in the microcirculation underlying the parietal peritoneum. However, these *in vivo* results indicate that syndecan-1 is localized to the basolateral side of the endothelial cell layer of venules, challenging the idea that syndecan-1 is part of the glycocalyx lining the luminal surface of the vasculature. Venular endothelial cells are surrounded by a basement membrane with interspersed pericytes. Syndecan-1 is known to bind various extracellular matrix components, such as collagen type IV [35], which is a major constituent of basement membranes. Thus, our findings are more consistent with the idea that syndecan-1 anchors the venular endothelial cells, and perhaps pericytes, to the underlying basement membrane. Since the present study is the first report of intravital imaging of syndecan-1 in the mouse microcirculation of the parietal peritoneum, it is possible that the different expression pattern observed is specific to this tissue.

Syndecan-1 is predominantly expressed on epithelial cells in a basolateral fashion. This basolateral restriction is dependent on the interactions of the PDZ-binding motif of the cytoplasmic tail of syndecan-1 with cytosolic PDZ domain-containing proteins [36], [37]. Since the mesothelial cells that form the parietal peritoneum are structured as squamous epithelium, we hypothesized that syndecan-1 is also expressed on these cells. Indeed, the fluorescent *ex vivo* images of the transverse sections of the anterior abdominal wall showed that syndecan-1 is expressed on the mesothelial cells that form the parietal peritoneum.

Cell surface and plasma levels of syndecan-1 change in inflammation and other pathological conditions. During hemorrhagic shock, plasma levels of shed syndecan-1 in severely injured patients were increased [38]. Systemic shed syndecan-1 levels were also reported to be associated with inflammation, coagulopathy and increased mortality in trauma patients [39]. The significance of syndecan-1 shedding is largely unclear, although it is postulated to mediate processes such as resolution of inflammation [23] and pathogen dissemination [40]. By quantifying the intensity from the fluorescent antibodies bound to syndecan-1 along the subendothelial surface, we showed that there is no significant difference in the level of syndecan-1 expression between LTA-treated animals and saline controls. While this suggests that syndecan-1 is constitutively expressed on the endothelium in the parietal peritoneum microcirculation and that no significant shedding occurs in inflammation, pro-inflammatory effects of the surgical preparation for IVM cannot be excluded. Thus, syndecan-1 levels in the tissue homogenate of the parietal peritoneum with the attached abdominal wall, as well as blood plasma and peritoneal lavage fluid were measured in response to IP injection of saline, LTA and TNF*α* by ELISA in a separate group of animals. In agreement with the fluorescence IVM results, the ELISA did not reveal any significant differences in syndecan-1 levels in the musculoperitoneal tissue, in the peritoneal effluent and in plasma between saline controls and LTA or TNF*α*-stimulated animals. These results suggest that inflammation did not promote syndecan-1 shedding. However, it is important to note that syndecan-1 shedding was repeatedly shown to be induced by pathogens [41], [42], such as *S. aureus* [8], and the microbial components that activate syndecan-1 shedding from cellular membranes were deduced to be *S. aureus α*- and *β*-toxins [43]. Thus, perhaps we didn't observe significant syndecan-1 shedding in this model of LTA-induced peritonitis because of the absence of certain staphylococcal toxins.

To study the role of syndecan-1 in leukocyte-endothelial cell interactions in the parietal peritoneum during inflammation, the microcirculatory responses to *S. aureus* LTA, *E. coli* LPS and TNF*α* were examined. While stimulation with LTA and TNF*α* significantly increased leukocyte adhesion in the vasculature of the musculoperitoneal wall, leukocyte adhesion did not significantly differ after injection of LPS compared with control and LPS treatment decreased the number of rolling leukocytes. The decreased inflammatory response to LPS in the parietal peritoneum microcirculation was not due to an inability of this tissue to respond to LPS as murine peritoneal cells do express the molecules of the LPS signaling pathway—Toll-like receptor 4 (TLR4), CD14 and myeloid differentiation factor 2 (MD2) [44]. Moreover, this effect is likely due to leukocyte sequestration in the lungs and the liver after IP injection of LPS. It was previously reported that IP injection of LPS rapidly causes neutropenia and TLR4-dependent neutrophil accumulation in the lungs [45]. This neutrophil sequestration in the lungs was associated with reduced leukocyte trafficking to peripheral vascular beds. Specifically, leukocyte rolling in the cremaster post-capillary venules was significantly decreased [45], similar to what we observed in the parietal peritoneum microcirculation.

Local and systemic microcirculatory responses to LPS and LTA differ substantially 4 hours after administration of these bacterial products [46]. In the murine cremaster muscle, local administration of *E. coli* LPS with an intrascrotal injection resulted in significantly increased numbers of rolling, adherent and extravascular leukocytes whereas intrascrotal *S. aureus* LTA injection had no effect on leukocyte-endothelial cell interactions in this vascular bed. Systemic administration of LPS with an IP injection resulted in decreased leukocyte recruitment in the cremasteric microcirculation but IP injection of LTA, again, had no effect on leukocyte recruitment to the cremaster muscle [46]. The IP injection of the bacterial products in the current report is a unique situation as this is both, a systemic response and a local response and, unlike the cremaster muscle, LTA injection had pro-inflammatory effects on the peritoneal microcirculation. Thus, even though the cremaster muscle is an extension of the abdominal musculature and its tunica vaginalis is a remnant of the peritoneum, there are significant differences in responses to LTA in the vascular beds of these tissues.

Our findings that *Sdc1* ^-/-^ mice have similar leukocyte recruitment to the wild-type controls during inflammation do not mirror observations in the retinal microcirculation as well as the mesenteric venules of *Sdc1* ^-/-^ mice [20]. *Sdc1* ^-/-^ mice on a BALB/c background were found to have an elevated number of adherent leukocytes in unstimulated retinal vasculature and bone marrow transplant studies indicated that the increased adhesion depended on *Sdc1* ^-/-^ leukocytes as opposed to the endothelial component [20]. The same study showed that 3 hours after IP injection of murine recombinant TNF*α*, *Sdc1* ^-/-^ mice had a significantly decreased number of rolling leukocytes and reduced rolling velocity. This was accompanied by a significantly increased number of adherent leukocytes per mesenteric venule and extravascular leukocytes were noted in the *Sdc1* ^-/-^ mice but not the wild-type controls. A consistent phenotype was observed by others in inflammatory and normal conditions. In murine experimental autoimmune encephalomyelitis, *Sdc1* ^-/-^ mice had increased infiltration by disease-causing Th1 and Th17 cells in brain tissue and increased disease severity [47]. Similarly, *Sdc1* ^-/-^ mice had increased and prolonged leukocyte infiltration in delayed-type hypersensitivity response [22]. In cremasteric venules of *Sdc1* ^-/-^ mice, there was increased leukocyte adhesion under normal conditions [48]. Also, syndecan-1 deficiency is marked by higher neutrophil and macrophage influx to the glomerulus in anti-glomerular basement membrane nephritis which was accompanied by increased injury and worse renal function [49]. Together, these studies suggest that syndecan-1 is a negative regulator of leukocyte recruitment and in the absence of syndecan-1, leukocyte recruitment is exaggerated and inflammatory reactions are dysregulated. The disparate findings between these studies and the current report may be due to differences in mechanisms that regulate inflammatory processes in the different tissues examined. Interestingly, *in vitro* work suggested that there is a greater increase in adhesion to endothelial culture cells with monocytes than neutrophils from *Sdc1* ^-/-^ mice [50]. However, we did not observe any CX_3_CR1^+^ monocyte recruitment to peritoneal venules 4 hours after saline, LTA or TNF*α* injection and the leukocyte infiltrate was composed of Ly6G^+^ neutrophils. Our findings suggest syndecan-1 is not a regulator of neutrophil recruitment during LTA-induced inflammation in the parietal peritoneum microcirculation during the early inflammatory response.

Syndecan-1 was shown to mediate neutrophil transendothelial migration *in vitro* [51]. To determine whether *Sdc1* ^-/-^ mice have altered transendothelial leukocyte migration, extravascular cells were counted. The number of extravascular leukocytes did not differ between the wild-type and *Sdc1* ^-/-^ treated animals after saline, LTA or LPS injection but was significantly increased in the *Sdc1* ^-/-^ mice with TNF*α* treatment, suggesting different mechanisms govern leukocyte extravasation with TNF*α*. In fact, leukocyte recruitment in the hepatic microcirculation is governed by different molecular mechanisms depending on the pro-inflammatory stimulus and microvessel type [52], [53].

Since we did not observe syndecan-1 to regulate leukocyte recruitment in the peritoneal venules, we examined the role of other adhesion molecules in leukocyte rolling and adhesion in the microcirculation underlying the parietal peritoneum. We showed that leukocyte rolling in this tissue is mostly mediated by P-selectin with some dependency on *β*_2_ integrin. However, blockade of the adhesion molecules *β*_2_ integrin, ICAM-1 and VCAM-1 did not decrease leukocyte adhesion in the peritoneal microcirculation even though we showed that these molecules are expressed in the peritoneal venules. This suggests that there are redundant mechanisms that mediate leukocyte adhesion in this tissue.

Rolling leukocytes along the endothelial wall are exposed to chemokines on the luminal side and this promotes directional intravascular crawling and firm adhesion of the leukocyte to the endothelial cell which is ultimately followed by extravasation through the vessel wall [54]. Numerous studies reported that syndecan-1 binds to and concentrates chemokines in tissues. Syndecan-1 was found to be complexed with several different chemokines, including interleukin-8 (IL-8; CXCL8) [54], KC [10], RANTES (CCL5) and MCP-1 [9], as well as CCL7, CCL11 and CCL17 [21]. MIP-2 binding to the venular endothelium of the cremaster muscle was found to be dependent on the presence of heparan sulfate [54] and the chemokine-binding domain of heparan sulfate that associates with IL-8 was deciphered [55]. Chemokine binding to heparan sulfate chains was proposed to direct intraluminal crawling of neutrophils [54]. Yet in other situations, syndecan-1 binding to chemokines aided resolution of inflammation by removing MIP-2 and KC with syndecan-1 shedding [23].

For leukocyte extravasation to occur, a chemokine gradient across the vessel wall with higher chemokine concentrations in the extravascular space is required [54]. It was demonstrated that there is a steep gradient with heparan sulfate deposited on venules between the luminal and basolateral endothelial compartments, with higher heparan sulfate densities in the basolateral region [56]. This heparan sulfate distribution was suggested to support steep chemokine gradients between the luminal and subendothelial aspects of post-capillary venules. Given that syndecan-1 possesses heparan sulfate chains and is expressed along the subendothelial surface in the parietal peritoneum microcirculation, we hypothesized that it concentrates chemokines at the site of inflammation to direct extravasating leukocytes. Co-injection of fluorescent anti-syndecan-1 with anti-MIP-2 antibodies after LTA challenge revealed that syndecan-1 did partially co-localize with MIP-2 *in vivo*. However, *Sdc1* ^-/-^ mice had similar expression patterns of MIP-2 in the peritoneal venules, indicating that syndecan-1 is not necessary for MIP-2 deposition in the parietal peritoneum microcirculation. Interestingly, the anti-MIP-2 antibody binding pattern on the peritoneal venules was irregular, unlike the bladder [57] and liver microcirculation (unpublished observations), which exhibit a more uniform binding pattern of the antibodies to chemokines along the endothelial surface. Syndecan-1 may have a redundant role in chemokine deposition on venules with other heparan sulfate proteoglycans. We observed that syndecan-2 is also expressed on peritoneal venules with some overlap with MIP-2. However, the necessity of syndecan-2 for chemokine presentation on venules would be difficult to test *in vivo* since syndecan-2 null mice are not viable.

This study demonstrated that syndecan-1 is highly expressed along the subendothelial surface and the mesothelial cells of the parietal peritoneum. We showed that syndecan-1 does not regulate leukocyte recruitment during inflammation in the parietal peritoneum vasculature and that it is not necessary for chemokine presentation on the venular endothelium. These findings suggest that syndecan-1 is not a significant modulator of inflammatory responses in the parietal peritoneum microcirculation.
